# Annotations

**Published:** 1897-03-20

**Authors:** 


### ANNOTATIONS.

### To Drug or Not to Drug.

SOME people have a morbid fear of taking physic. They will consult the doctor readily enough, and pay him his fee ungrudgingly; but if they are asked to take medicine rather freely they wonder whether it may not, on a wide calculation, do them more harm than good; and they often end by not taking it, or by taking it too intermittently, or for too short a time for it to be of any real service to them. Now this is a survival from an earlier period, and a survival which does so much harm that it ought to be exposed and extirpated. It is easy to understand why our grandfathers and grandmothers had a wholesome fear of physic; and why they took as little of it as was compatible with obedience to the art of their times. They believed that the average doctor, on the one hand, followed no scientific rules in his practice, and on the other had a direct pecuniary interest in sending in as many physic bottles as possible. Human nature being what it then was, the grandfathers of our patients saw as little of the doctor and his physic as they possibly could. But new times new manners; and we may certainly also say, "new men." In our day, the doctor knows the physiological effects produced by his ordinary everyday medicines quite well; and, since he now often charges a fee for his visits and consultations, quite apart from his physic, there is little or no temptation for him to supply his patient with superfluous powders and pills. This general rule, then, for patients is, or ought to be, thoroughly established and trustworthy—whenever a doctor thinks it desirable to order physic, the patient ought loyally to take it. For this is a truism which no patient should forget, namely, that no medicine, prescribed by a competent doctor, can do so much harm to the patient as the disease can do. Is this the best which can be said for modern physic? By no means! It is merely an attempt, in a few lines, to deal with a common difficulty experienced by patients, and to offer two reasons which, taken together, ought to suffice for its removal.

### The Prison Treatment of Drunken Women.

AMONG men, as is well known, drink leads to crime. Among women, however, drink itself is the main crime. Not much more than ten per cent. of the female prison population is accounted for by offences against the person or the property of others, the prisons being filled to repletion with chronic female drunkards, who are slaves to the vice and are no sooner discharged from one short sentence than they return again as drunk as ever; and it becomes a very serious question indeed whether prisons are being put to their proper purpose when they are thus used as more or less permanent homes for chronic drunkards. Dr. Pitcairn, assistant surgeon to Holloway Prison, in a paper recently read by him on the prison treatment of inebriates, said that he could not remember to have met with more than ten or a dozen male drunkards whose convictions exceeded, or even reached, a score; but that the records of the police-courts conclusively showed that "once a drunkard always a drunkard" was almost an axiom in the case of females. The worst of it is that committal to prison soon ceases to have any terrors for these women, or to have any effect whatever in checking their downward career. A prison should at least be a place to dread; but in reality the prisoner finds herself treated with kindness, is warmly clad, fed amply, if plainly, works to a considerably less degree than when at liberty, and sleeps amid good and sufficient bedding in a warmed and ventilated chamber. What is there deterrent in this, when a woman once has fallen so as not to feel ashamed? The matter is greatly aggravated by the fact that so many of these women are actually ill in consequence of their drinking habits when arrested, and thus they are perforce treated as invalids throughout their entire sentence of "hard labour." The prison thus becomes a hospital for drunkards supported at the public expense. Nothing could be more calculated to bring the law into contempt than such a use of our prison system. Much that has been said about the necessity of legislation for inebriates has been founded upon the terrible social evils which accompany the unpunished drunkenness of men; but an equally good case could be founded on the fact that the punishment of drunken women is turning our prisons into mere drink hospitals, and destroying the deterrent influence of imprisonment.

### Writers or Fighters?

SOME glimmering of reason seems to be at last penetrating the general mind on the question of the competence of the merely literary test of the merit or fitness of all sorts and conditions of men for all sorts of callings. A correspondent in the *Times* writing on the all-important subject of the "physique of military officers," announces that the "literary test is valueless as a gauge of the moral and physical power which go to make leaders of men." He goes on to argue that "intellectual power is dearly bought at the expense of stamina and virility." He might, we think, have gone even further, and said that the capacity for passing examinations in literary subjects is for the most part dependent upon memory alone; and is, as a rule, no sure indication even of mental endowment. It is the case, in fact, that literary examinations alone in such practical subjects as fighting, doctoring, and even in "parsoning" and "lawyering," are a fraud and a delusion. The first of all necessities in the military officer is physique. It is stated that for every vacancy in our army there are five applicants. Among those five it is probable that at least three would be men of good physique. The practical thing to do would be to make the doctor the "agent of first instance" in the selection of officers. Let the standard of physical power and endurance be fixed at a high level, let us have an assurance that our officers can not only think well, but that they can see well, eat well, and digest well, and can "rough it" without suffering in health; and let all who are not "fit" in physique be decisively rejected before beginning any special reading for the army examinations. If there is so large a proportion as three physically fit candidates for every vacancy the literary tests can, after medical selection, be applied to the suitable three. Even then a literary test would not be of itself entirely competent. For leaders of other men, as the *Times* correspondent points out, we want men of high and strong morale. Indeed, one might almost say that if we could secure a high degree of moral and physical fitness the literary capacity among the better class of educated Englishmen might almost be left to take care of itself.

